# Long non-coding RNA AGAP2-AS1 exerts oncogenic properties in glioblastoma by epigenetically silencing TFPI2 through EZH2 and LSD1

**DOI:** 10.18632/aging.102018

**Published:** 2019-06-11

**Authors:** Wenzheng Luo, Xueyuan Li, Zhenyu Song, Xuqiang Zhu, Shanshan Zhao

**Affiliations:** 1 Department of Neurosurgery, The First Affiliated Hospital of Zhengzhou University, Zhengzhou 450052, Henan, P.R. China; 2 Department of Magnetic Resonance Imaging, The First Affiliated Hospital of Zhengzhou University, Zhengzhou 450052, Henan, P.R. China

**Keywords:** glioblastoma, lncRNA, AGAP2-AS1, TFPI2, EZH2

## Abstract

Long non-coding RNAs (LncRNAs) have attracted increasing attention for their important regulation functions in a wide range of malignancies. AGAP2-AS1 was demonstrated as an oncogene in several cancers, including glioblastoma (GBM). However, the biological mechanisms of AGAP2-AS1 in GBM progression are still unclear. Herein, we found that AGAP2-AS1 expression was up-regulated in GBM tissues and cells. High AGAP2-AS1 expression may predict a poor prognosis in GBM patients. Functionally, silencing of AGAP2-AS1 suppressed proliferation and invasion, while enhanced apoptosis in GBM cells. Overexpression of AGAP2-AS1 promoted cell proliferation and invasion. Mechanically, AGAP2-AS1 could interact with EZH2 and LSD1, recruiting them to TFPI2 promoter region to inhibit its transcription. Moreover, TFPI2 overexpression decreased proliferation and invasion, and facilitated apoptosis in GBM cells. Furthermore, the tumor-suppressive effects mediated by AGAP2-AS1 knockdown were greatly reversed following down-regulation of TFPI2. Also, suppression of AGAP2-AS1 impaired tumor growth of GBM *in vivo*. In summary, AGAP2-AS1 exerts oncogenic functions in GBM by epigenetically silencing TFPI2 expression through binding to EZH2 and LSD1, illuminating a novel mechanism of AGAP2-AS1 in GBM development and furnishing a prospective therapeutic method to combat GBM.

## INTRODUCTION

Glioblastoma (GBM) is the most common malignant primary brain tumor in adults and one of the most deadly types of human cancers [1]. In 2016, it is estimated that there are 12,120 newly diagnosed GBM patients in the United States, with a 5-year survival rate of only 5% [2]. In spite of the advancement of surgical intervention, radiation, and systemic chemotherapy, the prognosis for patients with GBM remains dismal, with a median survival of 15 months [3, 4]. Therefore, better understanding of the molecular mechanisms underlying GBM development is meaningful to improve the diagnosis and treatment of GBM.

Long non-coding RNAs (LncRNAs) are a kind of transcripts with lengths greater than 200 nucleotides and little protein-coding capacities. LncRNAs are reported to participate in diverse pathological events in human malignancies by modulating mRNA stability, RNA splicing, chromatin structure, and miRNA-mediated gene regulation [5, 6]. Increasing lncRNAs have been identified as oncogenes or tumor suppressors in different cancers, including glioma [7, 8].

Accumulating lncRNAs have been revealed to epigenetically silencing gene expression by binding to PRC2 (polycomb repressive complex 2) in various biological processes, especially in cancer [9, 10]. Enhancer of zeste homolog 2 (EZH2), a key catalytic subunit of the PRC2 (EZH2, SUZ12, and EED), serves as a histone methyltransferase to enhance histone H3 lysine 27 trimethylation (H3K27me3) and suppress gene expression [7]. EZH2 is found to be overexpressed and exert oncogenic activity in multiple cancer types [11]. Lysine-specific demethylase 1(LSD1) is a demethylase that mediates the enzymatic demethylation of H3K4me1/2 and results in transcriptional repression [12]. LSD1 facilitates human carcinogenesis through chromatin regulation in various cancers [13]. Mounting evidence suggests that lncRNAs could regulate cancer phenotypes via interaction with EZH2 and LSD1. For example, lncRNA PVT1 contributed to non-small cell lung cancer (NSCLC) progression by EZH2-medicated inhibition of the LATS2/MDM2/P53 pathway [14]. LncRNA FEZF1-AS1 epigenetically inhibited downstream gene p21 via binding to LSD1, thereby facilitating proliferation in advanced stages of gastric cancer [15]. LncRNA HOXA-AS2 promoted cell proliferation and decreased apoptosis in colorectal cancer through recruiting EZH2 and LSD1 to p21 and KLF2 promoter regions to repress their transcriptions [16].

AGAP2-AS1, an antisense lncRNA located at 12q14.1 and 1567 nt in length, was firstly found to be up-regulated and associated with poor prognosis of NSCLC [17]. AGAP2-AS1 was demonstrated as an oncogenic lncRNA in NSCLC [17] and gastric cancer [18] by interacting with LSD1 and EZH2. Moreover, AGAP2-AS1 expression was increased with tumor grade in anaplastic glioma, and knockdown of AGAP2-AS1 suppressed cell proliferation, migration and invasion, while promoted apoptosis *in vitro* [19]. However, the biological functions and molecular mechanisms of AGAP2-AS1 in GBM need to be further investigated.

In this study, we found that AGAP2-AS1 expression was increased in GBM tissues and cells. Additionally, down-regulation of AGAP2-AS1 impaired proliferation and invasion, while induced apoptosis of GBM cells. Furthermore, AGAP2-AS1 epigenetically inhibited TFPI2 expression by binding to EZH2 and LSD1, thus promoting GBM progression. Together, illuminating the roles and mechanisms of AGAP2-AS1 will provide novel insights for GBM therapy.

## RESULTS

### AGAP2-AS1 expression was up-regulated in GBM and positively correlated with poor prognosis

**Figure 1:**
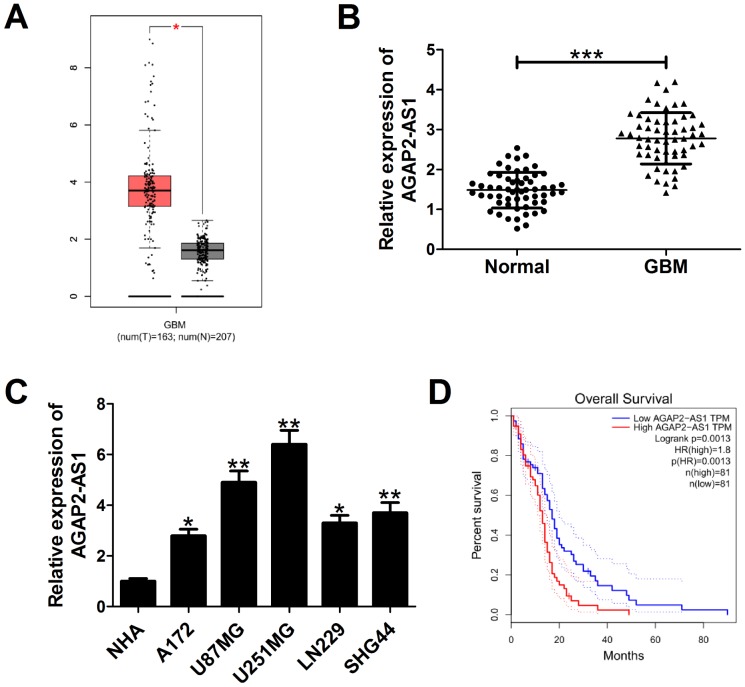
**AGAP2-AS1 expression is overexpressed in GBM and correlated with poor prognosis of GBM patients.** (**A**) AGAP2-AS1 expression in GBM tissues (n=163) and normal tissues (n=207) in from GEPIA database (http://gepia.cancer-pku.cn/detail.php?gene=&clicktag=boxplot). (**B**) qRT-PCR analysis of AGAP2-AS1 expression in tumor tissues and matched surrounding tissues from 58 patients with GBM. (**C**) qRT-PCR analysis of AGAP2-AS1 enrichment in five GBM cells (A172, U87/MG, U251/MG, LN229, SHG44) and normal human astrocytes (NHA). (**D**) Kaplan-Meier analysis of overall survival in GBM patients according to AGAP2-AS1 expression levels. *P < 0.05, **P < 0.01, ***P < 0.001.

According to the data from bioinformatics tool GEPIA (http://gepia.cancer-pku.cn/detail.php?gene=&clicktag=boxplot), AGAP2-AS1 expression was higher in 163 GBM tissues than that in 207 normal tissues (Figure 1A). In order to validate this result, qRT-PCR was performed to measured AGAP2-AS1 expression in 58 paired GBM tumor tissues and adjacent normal tissues. The results showed an increased AGAP2-AS1 expression in tumor tissues versus matched histologically non-cancerous tissues (Figure 1B). Also, we detected endogenous expression of AGAP2-AS1 in various GBM cell lines (A172, U87/MG, U251/MG, LN229, SHG44) and normal human astrocytes (NHA). As expected, AGAP2-AS1 expression was significantly up-regulated in GBM cells when compared with NHA (Figure 1C). Moreover, GEPIA data displayed that patients with high AGAP2-AS1 expression had a shorter overall survival than those with low AGAP2-AS1 level (Figure 1D). All these results indicated that AGAP2-AS1 was up-regulated and associated with poor prognosis in GBM.

### Knockdown of AGAP2-AS1 suppressed proliferation and invasion, and facilitated apoptosis in GBM cells

**Figure 2:**
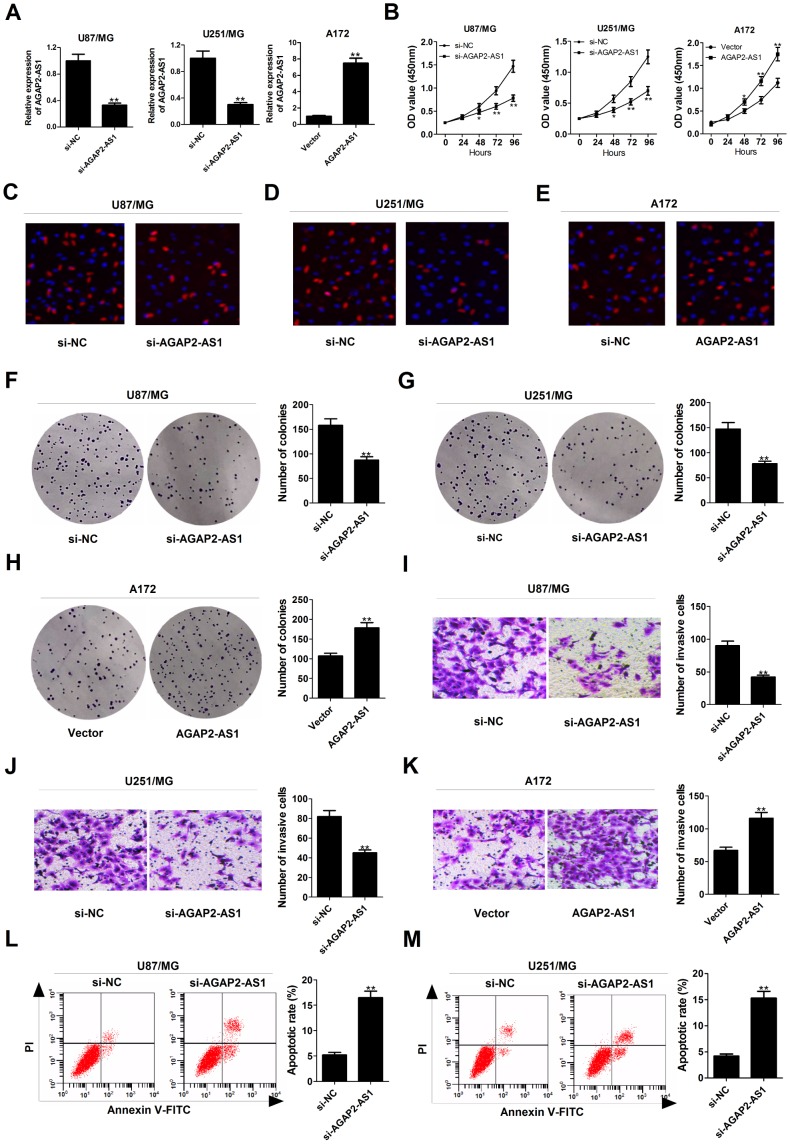
**Silencing of AGAP2-AS1 represses proliferation and invasion, and promoted apoptosis in GBM cells.** U87/MG and U251/MG cells were transfected with si-NC or si-AGAP2-AS1, while A172 cells were introduced with empty vector or pcDNA-AGAP2-AS1. (**A**) qRT-PCR analysis of transfection efficiency in U87/MG, U251/MG and A172 cells. (**B**) CCK-8 analysis was conducted to detect the viability in transfected U87/MG, U251/MG and A172 cells. (**C**–**E**) (d) EdU staining assay was performed to evaluate the proliferation ability in transfected U87/MG, U251/MG and A172 cells. Red, EdU staining for dividing cell; blue, DAPI staining for nuclear. (**F**–**H**) Colony-forming assay was used to examine the cloning ability in transfected U87/MG, U251/MG and A172 cells. (**I**–**K**) Transwell assay was carried out to assess the invasiveness in transfected U87/MG, U251/MG and A172 cells. (**L** and **M**) Flow cytometry analysis was applied to determine the apoptotic rate in transfected U87/MG, U251/MG and A172 cells. *P < 0.05, **P < 0.01.

To explore the functional relevance of AGAP2-AS1 in GBM cells, we interfered endogenous AGAP2-AS1 expression in U87/MG and U251/MG cells by transfection with specific siRNA, and increased AGAP2-AS1 expression in A172 cells by transfection with one overexpression plasmid (Figure 2A). CCK-8 assays showed that knockdown of AGAP2-AS1 impaired the growth ability of U87/MG and U251/MG cells, whereas overexpression of AGAP2-AS1 promoted the proliferation capability of A172 cells (Figure 2B). EdU staining assay manifested that the proliferation potential was suppressed in U87/MG and U251/MG cells following down-regulation of AGAP2-AS1 (Figure 2C and 2D), while AGAP2-AS1 up-regulation increased A172 cell proliferation (Figure 2E). Colony formation assay demonstrated that the clonogenic ability was reduced in U87/MG and U251/MG cells after inhibiting AGAP2-AS1 expression (Figure 2F and 2G), but was enhanced in AGAP2-AS1-overexpressing A172 cells (Figure 2H). Transwell assays presented that depletion of AGAP2-AS1 resulted in a suppression of invasive ability in U87/MG and U251/MG cells (Figure 2I and 2J), while a promotion of invasiveness in AGAP2-AS1-transfected A172 cells (Figure 2K). Flow cytometry assays also revealed that silencing of AGAP2-AS1 greatly induced apoptosis in U87/MG and U251/MG cells (Figure 2L and 2M). All these data suggested the oncogenic role of AGAP2-AS1 in GBM progression *in vitro*.

### AGAP2-AS1 inhibited TFPI2 transcription by binding with LSD1 and EZH2 in GBM cells.

**Figure 3:**
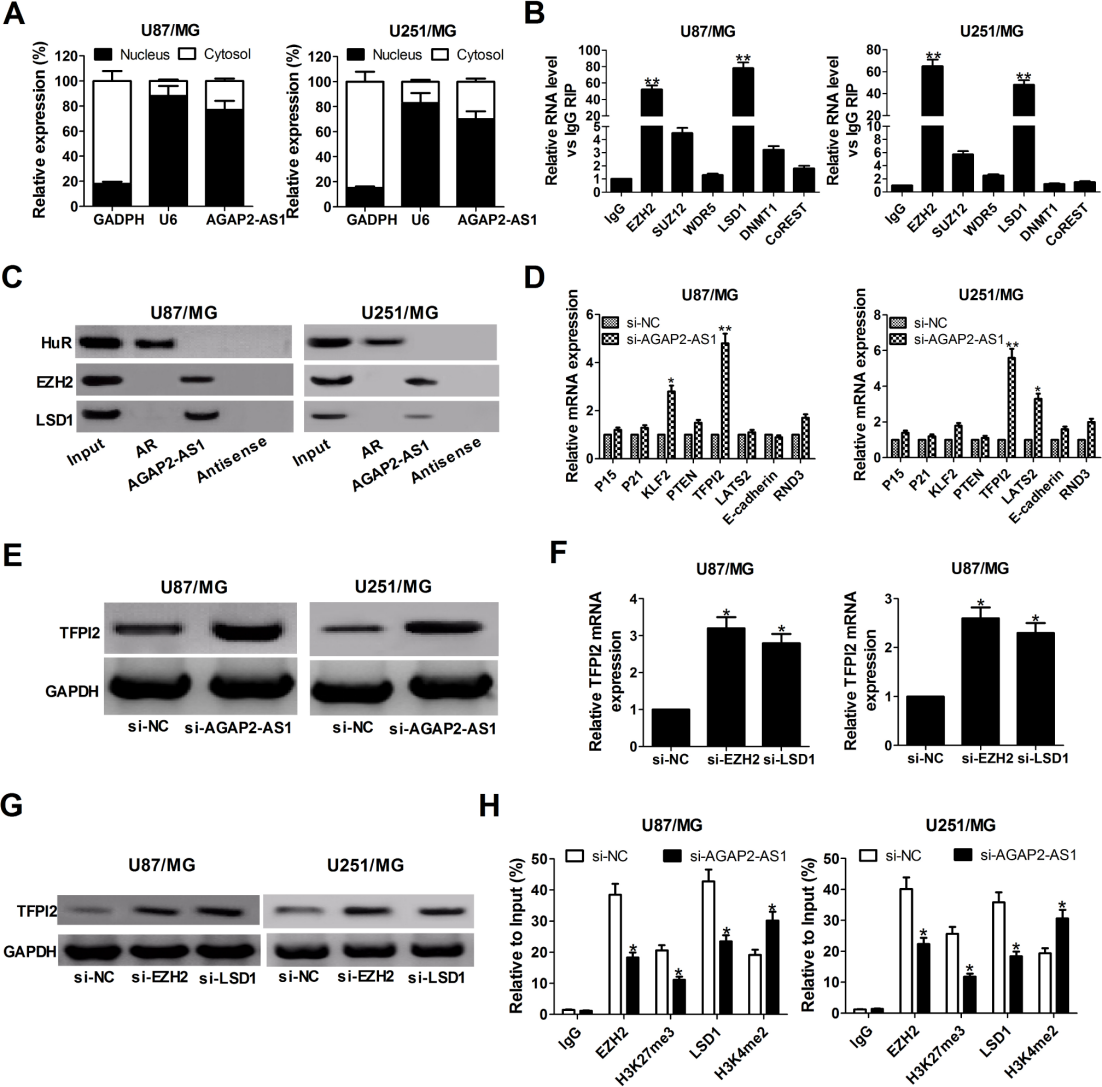
**AGAP2-AS1 recruits EZH2 and LSD1 to suppress TFPI2 expression.** (**A**) qRT-PCR analysis of AGAP2-AS1 level in the nuclear and cytoplasmic fraction of U87/MG and U251/MG cells. GAPDH and U6 were used as the cytoplasm marker and nucleus marker, respectively. (**B**) RIP experiments were conducted in U87/MG and U251/MG cells using antibodies against EZH2, SUZ12, WDR5, LSD1, DNMT1, and CoREST, followed by qRT-PCR assay of AGAP2-AS1 levels in immunoprecipitates. The fold enrichment was relative to IgG immunoprecipitate. (**C**) HuR, EZH2, and LSD1 protein levels in immunoprecipitates with AGAP2-AS1 were determined in U87/MG and U251/MG cells by western blot. Androgen receptor (AR) RNA was used as a positive control for HuR protein. (**D**) qRT-PCR analysis was performed to evaluate the levels of P15, P21, KLF2, PTEN, TFPI2, LATS2, E-cadherin and RND3 in si-NC- or si-AGAP2-AS1-transfected U87/MG and U251/MG cells. (**E**) Western blot analysis of TFPI2 expression in U87/MG and U251/MG cells following knockdown of AGAP2-AS1. (**F** and **G**) TFPI2 expression level at mRNA and protein levels were detected in U87/MG and U251/MG cells after transfection with si-EZH2 or si-LSD1. (**H**) ChIP assay of EZH2/LSD1 occupancy and H3K27me3/H3K4me2 binding in the TFPI2 promoter in U87/MG and U251/MG cells after transfection with si-NC or si-AGAP2-AS1. Enrichment was quantified relative to input control. IgG was used as a negative control. *P < 0.05, **P < 0.01.

It is well known that lncRNAs are able to regulate cell phenotypes through interacting with specific RNA-binding proteins. To examine the potential biological mechanisms of AGAP2-AS1 involved in GBM cells, subcellular fractionation assays were performed to determine the distribution of AGAP2-AS1 in nuclear and cytoplasmic fractions in GBM cells. Results revealed that AGAP2-AS1 was mainly located in the nucleus of U87/MG and U251/MG cells (Figure 3A), indicating that AGAP2-AS1 may exert regulatory effects at transcriptional levels. Then, RIP assays were used to analyze the possible RNA-binding proteins of AGAP2-AS1 in GBM cells. As presented in Figure 3B, AGAP2-AS1 could directly bind with EZH2 and LSD1 in U87/MG and U251/MG cells. Moreover, RNA pull-down assays showed that EZH2 and LSD1 in the nuclear extract fraction of U87/MG and U251/MG cells were pulled down by labeled AGAP2-AS1 (Figure 3C), further confirming the binding between AGAP2-AS1 and EZH2 or LSD1.

Next, we selected several EZH2 or LSD1 potential targets (P15, P21, KLF2, PTEN, TFPI2, LATS2, E-cadherin, and RND3) with tumor-suppressive role, and hypothesized that some of which may be associated with AGAP2-AS1-mediated carcinogenicity. qRT-PCR results disclosed that TFPI2 expression was significantly up-regulated in both U87/MG and U251/MG cells when AGAP2-AS1 was knocked down (Figure 3D). Western blot analysis also clarified an increase of TFPI2 protein level in AGAP2- AS1-depleted U87/MG and U251/MG cells (Figure 3E). Meanwhile, knockdown of EZH2 or LSD1 resulted in en evident up-regulation of TFPI2 expression at mRNA and protein levels (Figure 3F and 3G). These results implied that TFPI2 might be a downstream mediator of AGAP2-AS1.

To further address whether AGAP2-AS1 inhibited TFPI2 expression through interacting with EZH2 and LSD1, ChIP analysis was conducted in U87/MG and U251/MG cells. Results illuminated that AGAP2-AS1 could recruit EZH2 and LSD1 to the TFPI2 promoter region, resulting in trimethylation of H3K27 or demethylation of H3K4 at this region (Figure 3H). However, silencing of AGAP2- AS1 lowered their binding ability and H3K27me3 or H3K4me2 modification (Figure 3H). In summary, AGAP2-AS1 epigenetically suppressed TFPI2 expression by recruiting EZH2 and LSD1 to their promoter region in GBM cells.

### Overexpression of TFPI2 decreased proliferation and invasion, and induced apoptosis in GBM cells

**Figure 4:**
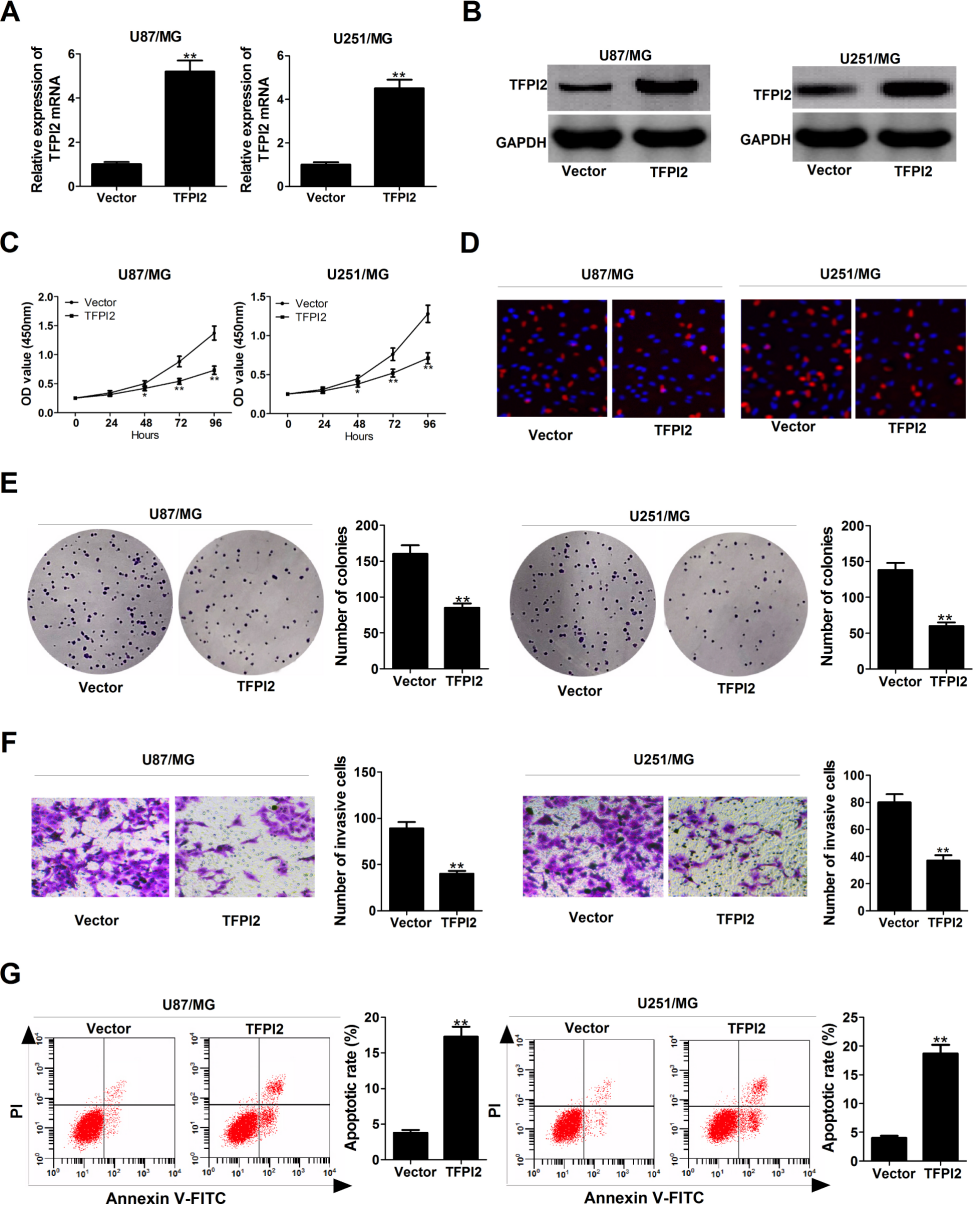
**TFPI2 up-regulation represses proliferation and invasion, and enhanced apoptosis in GBM cells.** (**A** and **B**) TFPI2 mRNA and protein levels were evaluated in U87/MG and U251/MG cells after transfection with empty vector or pcDNA-TFPI2. (**C** and **D**) CCK-8 and EdU staining assays were performed to examine the effects of TFPI2 overexpression on U87/MG and U251/MG cell proliferation. (**E**) Colony forming assay was conducted to detect the cloning ability in Vector- or pcDNA-TFPI2-transfected U87/MG and U251/MG cells. (**F**) Transwell assay of invasive ability in U87/MG and U251/MG cells following TFPI2 up-regulation. (**G**) Flow cytometry analysis of apoptotic rate in U87/MG and U251/MG cells introduced with empty vector or pcDNA-TFPI2. *P < 0.05, **P < 0.01.

Subsequently, we further explored the functions of TFPI2 in GBM cells by transfection with TFPI2-overexpression plasmid (TFPI2). As displayed in Figure 4A and 4B, TFPI2 mRNA and protein levels were obviously increased in U87/MG and U251/MG cells after transfection with pcDNA-TFPI2. CCK-8 and EdU staining assays demonstrated that up-regulation of TFPI2 led to a remarkable decline of proliferation ability in U87/MG and U251/MG cells (Figure 4C and 4D). Colony formation assay confirmed the suppressive effect of TFPI2 overexpression on clonogenic ability (Figure 4E). Transwell assay declared that enforced expression of TFPI2 dramatically reduced the invasive ability of U87/MG and U251/MG cells (Figure 4F). Flow cytometry analysis highlighted a significant increase of apoptotic rate in U87/MG and U251/MG cells upon TFPI2 overexpression (Figure 4G). Together, TFPI2 inhibited cancer phenotype in GBM *in vitro*.

### AGAP2-AS1 exerts oncogenic activity by silencing TFPI2 in GBM cells

**Figure 5:**
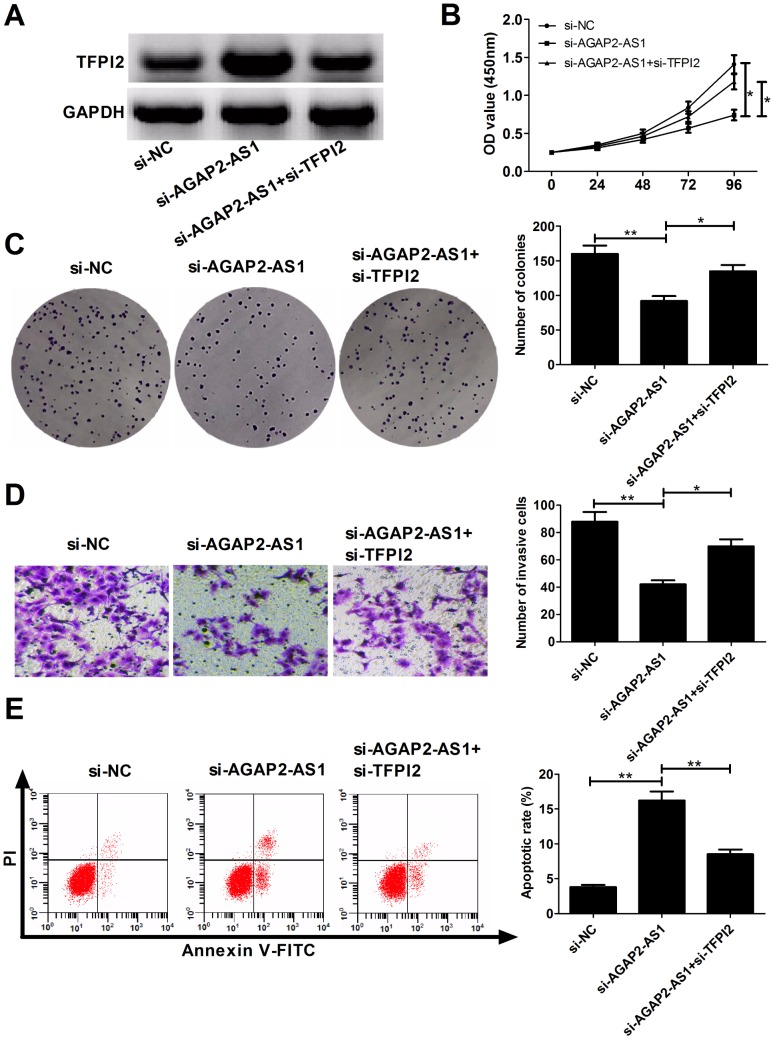
**AGAP2-AS1 promotes GBM progression partially through epigenetically inhibiting TFPI2 expression *in vitro*. U87/MG cells were transfected with si-NC, si-AGAP2-AS1, or co-transfected with si-AGAP2-AS1 and si-TFPI2.** (**A**) Western blot analysis of TFPI2 protein level in transfected cells. (**B**) CCK-8 assay of proliferation ability in transfected cells. (**C**) Colony formation assay of cloning ability in transfected cells. (**D**) Transwell assay of invasiveness in transfected cells. (**E**) Flow cytometry analysis of apoptosis in transfected cells. *P < 0.05, **P < 0.01.

To gain insight into whether TFPI2 was involved in AGAP2-AS1-mediated oncogenic functions, rescue experiments were conducted in U87/MG cells by co-transfection with si-AGAP2-AS1 and si-TFPI2. Results revealed that si-AGAP2-AS1-induced increase of TFPI2 expression was greatly abrogated by TFPI2 knockdown in U87/MG cells (Figure 5A). Moreover, silencing of TFPI2 partly reversed the anti-proliferation (Figure 5B and 5C), anti-invasion (Figure 5D) and pro-apoptosis (Figure 5E) effects caused by AGAP2-AS1 knockdown. These data suggested that AGAP2-AS1 contributed to GBM progression possibly by down-regulating TFPI2.

### Knockdown of AGAP2-AS1 inhibited GBM tumorigenesis *in vivo*

**Figure 6:**
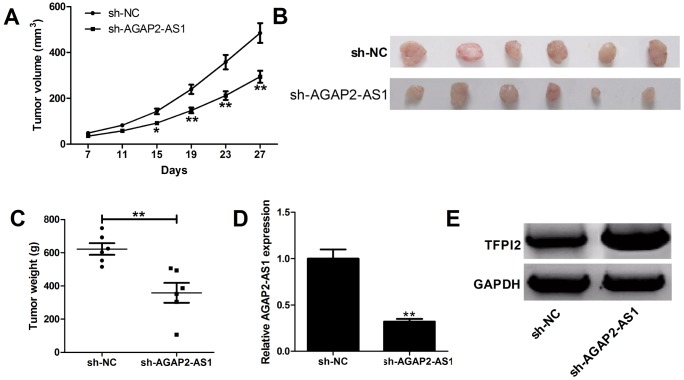
**Silencing of AGAP2-AS1 suppresses GBM growth *in vivo*. U87/MG cells stably transfected with sh-NC or sh-AGAP2-AS1 were inoculated into nude mice (n=6).** (**A**) Tumor volumes were monitored from the seventh day after cell injection. (**B** and **C**) At day 27 after cell inoculation, the tumors were removed, photographed and weighed. (**D**) qRT-PCR analysis of AGAP2-AS1 expression in tumors. (**E**) Western blot analysis of TFPI2 expression in tumors. *P < 0.05, **P < 0.01.

To further address the biological significance of AGAP2-AS1 in tumor growth *in vivo*, sh-NC or sh-AGAP2-AS1 stably transfected U87/MG cells were subcutaneously injected into the left flank of nude mice. Results showed that silencing of AGAP2-AS1 obviously slowed down the tumor growth (Figure 6A). Moreover, the weights were decreased in tumors developed from sh-AGAP2-AS1-transfected U87/MG cells (Figure 6B). Additionally, knockdown of AGAP2-AS1 triggered a reduction of AGAP2-AS1 expression (Figure 6D), while an increase of TFPI2 protein level (Figure 6E) in excised tumor masses. In all, depletion of AGAP2-AS1 impaired tumor xenograft possibly by modulating TFPI2.

## DISCUSSION

It is widely accepted that lncRNAs affect cancer phenotypes by modulating target gene expression via various mechanisms, such as chromatin modification, genomic imprinting, RNA decay and sponging miRNAs [20]. To date, accumulating lncRNAs have been identified as critical players in regulating cell proliferation, apoptosis, genomic instability, drug resistance, invasion, and metastasis in a range of malignant tumors, including glioma [21, 22]. A previous report demonstrated that AGAP2-AS1 expression was up-regulated with tumor grade in anaplastic glioma, and depletion of AGAP2-AS1 inhibited cancer cell progression *in vitro* [19]. Herein, we focused on explaining the functional roles and potential mechanisms in GBM development.

Firstly, we found that AGAP2-AS1 expression was increased in GBM tissues and cells. Moreover, higher AGAP2-AS1 expression predicted a poor clinical outcome in GBM patients, in accordance with the report by Tian *et al*. [23]. Then, we characterized the role of AGAP2-AS1 in GBM by using loss- and gain-of-function assays. Results showed that down-regulation of AGAP2-AS1 blocked proliferation and invasion, and promoted apoptosis in U87/MG and U251/MG cells, while overexpression of AGAP2-AS1 displayed the contrary effects in A172 cells. Consistently, Li *et al*. revealed that AGAP2-AS1 up-regulation resulted in increased proliferation, migration and invasion, and decreased apoptosis in NSCLC cells [17]. Qi *et al*. proved that AGAP2-AS1 overexpression induced cell proliferation and invasion in gastric cancer [18]. Dong *et al*. displayed that AGAP2-AS1 contributed to breast cancer growth and trastuzumab resistance [24]. Moreover, a recent document confirmed that silencing of AGAP2-AS1 depressed cell proliferation, migration, and invasion, and enhanced apoptosis in GBM [23]. Nevertheless, the regulatory mechanisms of AGAP2-AS1 in GBM are still unknown.

To our knowledge, one of the mechanisms for lncRNAs is to recruit proteins or RNAs to target genes, thus exerting biological significance indirectly [25]. To clarify the possible mode of action of AGAP2-AS1 involved in GBM pathogenesis, subcellular fractionation assays were performed. As a result, AGAP2-AS1 was found to be mostly located in the nucleus fractions of U87/MG and U251/MG cells, reflecting its regulation at transcriptional level. Subsequent RIP and RNA pull down assays verified that AGAP2-AS1 could directly bind to EZH2 and LSD1. Aberrant high expressions of EZH2 and LSD1 have been reported to be associated with multiple cell processes in many malignancies [26, 27]. By using qRT-PCR and western blot analysis, we found an increase of TFPI2 mRNA and protein levels in U87/MG and U251/MG cells following knockdown of AGAP2-AS1. Similarly, elevated TFPI2 mRNA and protein levels were observed in EZH2- and LSD1-knockdown U87/MG and U251/MG cells. That is to say, TFPI2 is a novel AGAP2-AS1 target in GBM cells. EZH2 is a negative regulator of transcription via histone 3 lysine 27 trimethylation (H3K27me3), whereas LSD1 is a negative regulator of transcription via histone 3 lysine 4 demethylation (H3K4me2). Further ChIP assays elucidated that AGAP2-AS1 could recruit EZH2 and LSD1 to TFPI2 promoter region and suppress their transcription through mediating H3K27me3 and H3K4me2 modifications. To conclude, AGAP2-AS1 epigenetically silenced TFPI2 expression in GBM cells via binding to EZH2 and LSD1. In accordance with our results, AGAP2-AS1 was validated to exert oncogenic functions partly by suppressing the transcription of downstream targets by interacting with epigenetic proteins, such as EZH2, LSD1, and CBP (CREB-binding protein), in NSCLC [17], gastric cancer [18] and breast cancer [23].

Tissue factor pathway inhibitor 2 (TFPI2), a member of the Kunitz-type serine proteinase inhibitor family, has been deemed as a tumor suppressor in several neoplasms, such as bladder cancer [28], cholangiocarcinoma [29], small cell lung cancer [30], and breast cancer [31]. In the current study, overexpression of TFPI2 reduced proliferation and invasion, while facilitated apoptosis in GBM cells. Consistent with our data, George *et al*. disclosed that TFPI2 restoration induced both intrinsic and extrinsic caspase-mediated pathway, thereby facilitating apoptosis in a GBM cell line [32]. Gessler *et al*. discovered that TFPI-2 knockdown promoted proliferation, migration and invasion in glioma cells [33]. Yanamandra *et al*. revealed that recombinant adeno-associated virus (rAAV) expressing TFPI-2 abated invasion, angiogenesis and tumor growth in a human GBM cell line [34]. Moreover, TFPI2-induced cell apoptosis was associated with lncRNA AC003092.1-mediated TMZ sensitivity in GBM [35]. Furthermore, we found that si-AGAP2-AS1-elicited anti-proliferation, anti-invasion and pro-apoptosis effects were substantially reversed following TFPI2 knockdown. Finally, depletion of AGAP2-AS1 impaired GBM tumor growth *in vivo* possibly through increasing TFPI2 expression.

In conclusions, our study showed that AGAP2-AS1 expression was up-regulated in GBM tissues and cells. silencing of TFPI2 expression via acting as a scaffold for EZH2 and LSD1. Our study provides a novel Functionally, AGAP2-AS1 knockdown inhibited proliferation and invasion, and induced apoptosis in GBM cells. Mechanically, AGAP2-AS1 exerted oncogenic activity partially through epigenetic insight into the mechanisms of GBM tumorigenesis, and highlights a promising molecular target for GBM patients. However, it is worth to further explore the other possible regulatory mechanisms of AGAP2-AS1 involved in GBM progression.

## MATERIALS AND METHODS

### Patients and tissue samples

GBM tumor tissues and matched non-cancerous tissues were obtained from 58 GBM patients diagnosed by experienced pathologists at the Department of Neurosurgery at the First Affiliated Hospital of Zhengzhou University. No local or systemic treatment was administrated to patients before surgery. Fresh specimens were immediately snap frozen in liquid nitrogen and then stored at -80°C. The protocol used in this study was approved by the Research Ethic Committee of the First Affiliated Hospital of Zhengzhou University. All participants provide written informed consents prior to this study.

### Cell lines and culture conditions

GBM cell lines (A172, U87/MG, U251/MG, LN229, and SHG44) and normal human astrocytes (NHA) were purchased from the Institute of the Chinese Academy of Sciences (Shanghai, China). All cells were cultured in RPMI 1640 medium (Invitrogen, Carlsbad, CA, USA) containing 10% FBS at 37°C with 5% CO_2_.

### Transfection

To overexpress AGAP2-AS1 or TFPI2, the full length cDNA sequences of AGAP2-AS1 or TFPI2 were amplified and subcloned into pcDNA3.1 vector (Invitrogen) according to the manufacture’s guidelines. To suppress gene expression, small interfering RNAs (siRNAs) including si-AGAP2-AS1, si-EZH2, si-LSD1 and si-TFPI2 were purchased from RiboBio (Guangzhou, China). GBM cell were seeded into six-well plates and transfected with plasmids, siRNAs or corresponding negative control (Vector or si-NC) using Lipofectamine 2000 (Invitrogen).

### RNA extraction and qRT-PCR

Total RNA was extracted from frozen tissues and cultured cells using TRIzol reagent (Invitrogen) according to the manufacture’s protocol. Then, 1 μg of RNA was reversely transcribed into cDNA using QuantiTect Reverse Transcription Kit (Qiagen, Hilden, Germany). qRT-PCR reaction was performed using SYBR Green Master Mix (TaKaRa Bio, Otsu, Japan) on an ABI Prism 7900 Sequence Detection System (Applied Biosystems). The primer sequences used were listed as follows: AGAP2-AS1, 5′-TACCTTGACCTTGCTGCTCTC-3′ (Forward) and 5′-TGTCCCTTAATGACCCCATCC-3′ (Reverse); TFPI2, 5′-CTGGGGCTGTCGATTCTGC-3′ (Forward) and 5′-TCTCCGCGTTATTTCCTGTTG-3′ (Reverse); GAPDH, 5′-CGCTCTCTGCTCCTCCTGTTC-3′ (Forward) and 5′-ATCCGTTGACTCCGACCTTCAC-3′ (Reverse). The expression levels of AGAP2-AS1 and TFPI2 mRNA were normalized to GAPDH.

### Cell proliferation analysis

Cell proliferation was detected using a Cell Counting Kit-8 (CCK-8; Beyotime, Beijing, China) in accordance with the manufacture’s specification. GBM cells were plated into 96-well plates after transfection and counted every 24 h. The optical absorbance at 450 nm was measured. Cell viability was also determined with the 5-ethynyl-2′-deoxyuridine (EdU) assay using Cell-light EdU Apollo 567 In Vitro Imaging Kit (Ribobio, Guangzhou, China) following the manufacturer’s instructions. For the colony formation assay, cells were plated in 6-well plates and cultured in medium with 10% FBS to allow colony formation. The medium was replenished every 4 days. Two weeks later, cells were fixed with 96% ethanol and stained with 0.1% crystal violet. Colony formation ability was evaluated by counting the number of colonies consisting of more than 50 cells.

### Transwell assay of cell invasion

Cell invasion assays were performed using a transwell chamber (Corning Incorporated, Corning, NY, USA) pre-coated with Matrigel (BD Biosciences, Franklin Lakes, CA, USA). Briefly, 1 × 10^5^ GBM cells in serum-free RPMI 1640 medium were seeded on the top side of upper chamber, while medium containing 10% FBS was added into the lower chamber. Following 24 h of incubation, cells on the upper membrane were removed with a cotton swab. Cells that passed through the membrane were fixed with methanol and stained with 0.1 % crystal violet.

### Flow cytometric analysis of cell apoptosis

FITC Annexin V Apoptosis Detection Kit (BD Biosciences) was used to stain GBM cells according to the manufacturer’s recommendations. Then, cell apoptosis was measured by a FACScan flow cytometry (BD Biosicences).

### Western blot analysis

Protein lysates were separated by 10% SDS-PAGE gel, transferred to 0.22 μm nitrocellulose (NC) membrane (Millipore, Bedford, MA, USA), and then incubated with primary antibodies against EZH2 (Cell Signaling Technology, Beverly, MA, USA), LSD1 (Cell Signaling Technology), TFPI2 (Abcam, Cambridge, MA, USA) and GAPDH (Cell Signaling Technology). Immunoreactive bands were visualized by chemiluminescence, and protein expression was normalized to that of GAPDH.

### Subcellular fractionation location

PARIS Kit (Life Technologies, Carlsbad, CA, USA) was used to separate the nuclear and cytosolic fractions of GBM cells according to the manufacturer’s instructions. The levels of AGAP2-AS1, GAPDH and U6 RNA in cytoplasm and nuclear fraction were measured using qRT-PCR assay. GAPDH was used as the cytoplasm control, and U6 was used as the nuclear control.

### RNA immunoprecipitation (RIP)

RIP assay was performed to investigate whether AGAP2-AS1 could interact with the potential binding protein (EZH2, LSD1, and SUZ12 et al.) in GBM cells using Magna RIP™ RNA-Binding Protein Immunoprecipitation Kit (Millipore). Cells were scraped off and lysed in complete RIP lysis buffer. Then, the whole cell extract was incubated with magnetic beads coupled with specific antibodies or control IgG (Cell Signaling Technology). Subsequently, beads were washed and incubated with Proteinase K to remove proteins. Finally, the co-precipitated RNAs were purified and analyzed by qRT-PCR.

### RNA pull down assays

AGAP2-AS1 and antisense-AGAP2-AS1 were *in vitro* transcribed with TranscriptAid T7 High Yield Transcription Kit (Thermo Fisher Scientific, Waltham, MA, USA), purified with RNeasy Plus Mini Kit (Qiagen, Hilden, Germany), and then treated with RNase-free DNase I (Qiagen). Purified RNAs were biotin-labeled using Biotin RNA Labeling Mix (Roche Diagnostics, Indianapolis, IN, USA). Subsequently, biotinylated RNAs were mixed with magnetic beads and incubated with cell lysates. Finally, the beads were washed, and the eluted proteins were analyzed by western blot.

### Chromatin immunoprecipitation (ChIP)

ChIP assays were performed using the MagnaChIP Kit (Millipore) following the manufacturer's protocol. Cells were cross-linked with 1% formaldehyde, lysed and sonicated to acquire the DNA fragments of 200-500 bp. Then, the chromatin was immunoprecipitated with antibodies against EZH2, LSD1, H3K27me3, H3K4me2 or control IgG (Cell Signaling Technology). Precipitated chromatin DNA was purified and detected by qRT-PCR. The primer sequences for TFPI2 promoter region amplification are 5′-GGATGTTTGTTTTGTATAAAGTG-3′ (Forward) and 5′-AAACATCCAAAAAAACACCTAAC-3′ (Reverse). ChIP data is shown as a percentage relative to input DNA.

### Tumor xenograft model

Five-week-old male BALB/C nude mice were purchased from the Shanghai Sippr/BK Laboratory Animal Co. (Shanghai, China). U87/MG cells with nontarget sh-NC or sh-AGAP2-AS1 stable transfection were subcutaneously injected into the left flank of mice. Tumor growth was assessed every 4 days, and tumor volumes were calculated using the following formula: 0.5 × length × width^2^. After 27 days, the mice were sacrificed, and the tumors were removed, photographed and weighed. Tissue masses were also harvested for qRT-PCR, and western blot. This study complied with the National Institutes of Health Guide for the Care and Use of Laboratory Animals. The protocol was approved by the Ethics Committee of Animal experiments of the First Affiliated Hospital of Zhengzhou University.

### Statistical analysis

All experiments were conducted at least three times. All experimental data were presented as mean ± SD. The Student’s *t*-test or one-way ANOVA was performed to examine the difference between groups by using SPSS 20.0 software (SPSS, Chicago, IL, USA). *P* < 0.05 denotes a significant difference.
